# Biodiversity of mosquitoes and *Mansonia uniformis* as a potential vector of *Wuchereria bancrofti* in Hulu Sungai Utara District, South Kalimantan, Indonesia

**DOI:** 10.14202/vetworld.2020.2815-2821

**Published:** 2020-12-30

**Authors:** Muhammad Rasyid Ridha, Nita Rahayu, Budi Hairani, Dian Perwitasari, Harninda Kusumaningtyas

**Affiliations:** 1 Tanah Bumbu Unit for Health Research and Development, National Institute of Health Research and Development, National Ministry of Health of Indonesia; 2 Center of Research and Development Public Health Effort, National Institute Health Research and Development, Ministry of Health, Indonesia

**Keywords:** biodiversity, *Mansonia uniformis*, mosquito, *Wuchereria bancrofti*

## Abstract

**Background and Aim:**

Lymphatic filariasis, also known as elephantiasis, still remains a problem in Indonesia. The primary causative species of this disease are the filarial worms *Wuchereria bancrofti* and *Brugia* spp. This study was conducted to identify the diversity of species and behavior of mosquitoes and to determine the mosquitoes that could be potential vectors of filariasis.

**Materials and Methods:**

Mosquito samples derived from Hulu Sungai Utara (HSU) district in the 2017 multicenter study conducted in Indonesia were used in this cross-sectional study. The diversity of mosquito species was analyzed using the Shannon–Wiener diversity index. Mosquitoes were identified based on their species, and their DNA was isolated by polymerase chain reaction (PCR). Transcription-insulated isothermal PCR method was used to detect microfilariae/filaria larvae in the mosquitoes.

**Results:**

Biodiversity was found in 14 species of mosquitoes belonging to five genera. The maximum number of mosquitoes was recorded from the species *Mansonia dives*, *Culex vishnui*, *Culex quinquefasciatus*, and *Mansonia uniformis*. *W. bancrofti* infection was detected in *M. uniformis* at an infectivity rate of 0.3% (n=311).

**Conclusion:**

To the best of our knowledge, this is the first report of *M. uniformis* species as a vector of *W. bancrofti* in HSU district, Indonesia. More efficient and accurate studies are required to aid in the lymphatic filariasis elimination programs in this subregion.

## Introduction

Filariasis is a health problem in several countries of the world, including Indonesia [1]. Although filariasis rarely causes death, it can cause socioeconomic losses to the patients, which, in turn, decrease their productivity [2,3]. Until the end of 2016, a total of 236 filariasis endemic areas were recorded from 514 districts in Indonesia, one of which is Hulu Sungai Utara (HSU) district in South Kalimantan Province [4]. HSU district had implemented mass drug administration (MDA) from 2007 to 2011, but it had failed to evaluate the transmission assessment survey (TAS) in 2012, due to which it had to reimplement MDA. The failure in TAS evaluation at least indicates that the transmission of filariasis parasites still exists in that area.

Epidemiological transmission of filariasis involves several biological aspects, such as filarial worm agents, existence of vectors and reservoirs, environmental conditions of settlements, and all the socioeconomic and cultural aspects [5]. The types of lymphatic filariasis parasites found in Indonesia are *Wuchereria bancrofti*, *Brugia malayi*, and *Brugia timori*. The types of reservoir animals that are commonly infected with filarial worms are cats (*Felis catus*), dogs (*Canis familiaris*), and long-tailed monkeys (*Macaca fascicularis*) [6,7]. Mosquitoes belonging to the genera *Aedes*, *Anopheles*, *Coquillettidia*, *Culex*, and *Mansonia* are known as vectors of filariasis (depending on the geographic location). The existence of vectors in an area highly determines the level of filariasis transmission, especially the ability of mosquito vectors to ingest filarial worms and support their development into an infective stage (L3) after being in the mosquito’s body, and this depends on several influencing factors [8].

The prevention of filariasis in endemic areas involves the implementation of mass treatment and must be accompanied by integrated vector control measures. The HSU district had reimplemented MDA in 2014 and 2015, but it was not accompanied by vector control measures. Regarding the geographical conditions in the endemic villages of HSU district, they are generally surrounded by swampy waters and forests that are highly supportive of the life cycle and development of vector mosquitoes. Hence, it is extremely important to investigate the diversity of species and the behavior of mosquito vectors to determine effective and efficient control measures.

Therefore, this study was conducted to identify the diversity of species and the behavior of mosquitoes and to determine the mosquitoes that could be potential vectors of filariasis in the endemic villages of HSU district.

## Materials and Methods

### Ethical approval

This study approved by the Ethics Commission from National Institute Health Research and Development, Ministry of Health, Indonesia (No: LB.02.01/2/KE.167/2017). The research was a spot survey using a cross-sectional study [9].

### Study area and period

The research was conducted from July to September 2017 in the HSU district, an endemic area for *B. malayi* filariasis in South Kalimantan. Two villages, namely, Pihaung (2°22′44.38S 115°15′20.18″E) and Banjang (2°27′24.36″S 15°21′17.55″E), were surveyed with four mosquito captures for 5 months. The criterion for selecting the research area was the maximum number of filariasis-positive children assessed by peripheral blood examination using the Brugia Rapid Test™ in the area/village. The study population was all the existing mosquitoes, and the research sample consisted of mosquitoes captured during the study.

### Mosquito collection

**Figure-1:**
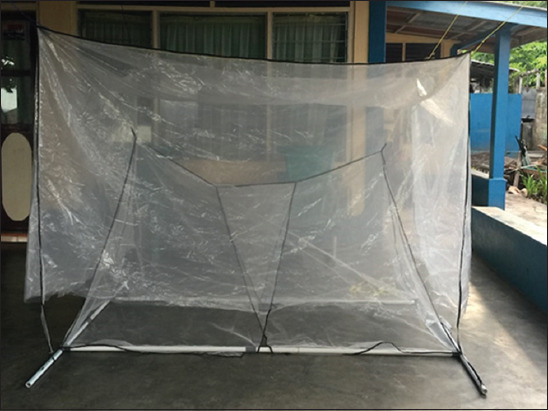
Human landing catches using a double net.

Mosquito samples were collected from selected areas in the 2017 multicenter study in Indonesia. The collection method was the modified human landing collection method inside a mosquito net, known as human landing catches using a double net (Figure-1) [10,11]. The method was implemented 2 times, with an interval of 1 month, at two sites/locations in each district/city for two consecutive nights, starting at 17:00 in the afternoon until the next day at 6.00 Indonesian zone time. Study location was the village where SDJ was implemented, selected at three location points, and collection was conducted in three houses inside and outside. For HSU district, there were two villages where the vector was to be collected. The outer mosquito net was in the form of a rectangle measuring 200×150×150 cm in size, and the inner mosquito net was in the form of a triangular prism measuring 150×90×120 cm in size. The outer mosquito net was left open at the bottom so that mosquitoes can enter the large net. The collector was placed on the inner small mosquito net. The collectors collected the mosquitoes that land either on the outside or inside the mosquito net every 10 min for 5 times. The mosquito catching period was 5 min after every 10 min.

Mosquito species were identified using a microscope and categorized as those that have already sucked blood (porous) and those that have never sucked blood (null). Chloroform was used for mosquito collapse to enable easy identification using a mosquito identification key [12]. Then, the mosquitoes were separated by taking the head and thorax and collected in a 1.5 mL microtube. Mosquitoes identified as same species were placed in a 1.5 mL microtube, with one microtube pool containing 1-25 mosquitoes [13].

### Detection of microfilariae in mosquitoes

Polymerase chain reaction (PCR) was conducted by pooling the mosquitoes according to species and location at the Centre of Public Health Efforts Laboratory of the Health Research and Development.

### Sample preparation

To the microtube containing 1-25 mosquitoes, 500 μL PBS was added. The mosquitoes were crushed using blue plastic sticks, and then centrifuged for 5 min. The supernatant was collected into a 1.5 mL microtube. This sample was now ready for DNA extraction.

### DNA extraction

The presence of microfilarial DNA was detected to observe traces of microfilarial DNA fragments in the mosquitoes. As a positive control, a positive sample collected from Musi Rawas District, South Sumatra Province, was used in this study. The collected mosquito species were identified, and their DNA was isolated using the transcription-insulated isothermal PCR (iiPCR) technique. The DNA from all samples was extracted using the Taco Preloaded DNA/RNA Extraction Kit on the Taco Mini Automatic Nucleic Acid Extraction System following the instructions provided by GeneReach. The supernatant (+ 500 μL) was collected into a 1.5 mL microtube, and taken 200 μL of supernatant to put into the holes in a Taco plate. The Taco plate was placed in the Taco machine according to the manufacturer’s instructions (running sample) for 25 min. The sample was taken out from the Taco kit pack, and a maximum of 150-200 μL of extracted RNA was collected into a 1.5 mL microtube (the remaining extracted RNA product was stored in a freezer at −40°C).

### PCR assay

Lyophilized premix pellets containing all primers, fluorescence hydrolysis probes, dNTP, and Taq DNA polymerases were used. PCR was conducted on a rapid PCR machine (GeneReach Biotechnology Corporation) using the forward HhaI primer (5′GCGCATAAATTCATCAGC-3′) and the reverse HhaII primer (5′GCGCAAAACTTAATTACAAAAGC-3′). Before the reaction, premix pellets were first dissolved in 50 μL premix buffer B and mixed with 5 μL DNA extract. The PCR premix was ready to use kit prepared by GeneReach Biotechnology Corporation. Next, 50 μL of the final reaction mixture was transferred to the R-tube for reaction in a rapid PCR machine. The device allows iiPCR to occur, collects and processes fluorescence signals automatically, and provides qualitative results (positive or negative) within 1 h. The default program includes two steps, viz., 50°C for 10 min (for reverse transcription, if needed) and 95°C for approximately 30 min (for iiPCR).

### PCR analysis

The signal-to-noise (S/N) ratio (fluorescence signal measured after iiPCR or fluorescence signal collected before iiPCR) was converted automatically into a plus (positive), minus (negative), or question mark according to the default S/N threshold set by the built-in algorithm. Qualitative results are shown on the display screen at the end of the program as + (positive) or − (negative). A question mark indicates that the result is ambiguous and that the sample needs to be tested again.

### Statistical analysis

The diversity of mosquito species was analyzed using the Shannon–Wiener diversity index, where a value of H′ ≤1 indicates low diversity, and a value of 1 ≤ H′ ≤ 3.00 indicates moderate diversity [14]. Entomological indices were calculated to determine relative abundance, species frequency, and species dominance [15].

## Results

### Species diversity, dominance, relative abundance, and dominance number

The Shannon–Wiener diversity index was generally low (0.201-<1). The highest diversity index of 0.4 was observed in the genus *Mansonia* in Pihaung and Banjang villages (Table-1). A total of 14 mosquito species belonging to five genera were found. The maximum number of mosquitoes were found in the species *Mansonia dives*, *Culex vishnui*, *Culex quinquefasciatus*, and *Mansonia uniformis*. *M. dives*, *M. uniformis*, and *C. vishnui* were generally found outdoors, whereas *C. quinquefasciatus* species were found more indoors (Table-2). Some of the mosquito species with the highest relative abundance were *M. dives* (24.6%), *C. vishnui* (22.8%), *C. quinquefasciatus* (18.5%), and *M. uniformis* (16.6%). All these species were always found in four catches so that the highest dominance number was also the four species (Table-3).

**Table-1:** Shannon–Wiener diversity index for genus caught in Hulu Sungai Utara District by human landing catches method.

Mosquito species	Shannon–Wiener diversity index		

	Pihaung village	Banjang village	Mean
*Aedes* spp.	0.1	0.0	0.2
*Anopheles* spp.	0.1	0.3	
*Coquelettidia* spp.	0.1	0.0	
*Culex* spp.	0.4	0.3	
*Mansonia* spp.	0.4	0.4	

**Table-2:** Species diversity of mosquitoes caught in Hulu Sungai Utara District by human landing catches method.

Mosquito species	Pihaung village		Banjang village		Total
	
	Indoors	Outhoors	Indoors	Outdoors	
*Aedes aegypti*	0	3	1	0	4
*Aedes albopictus*	2	6	0	0	8
*Coquelettidia crassipes*	8	9	0	1	18
*Coquelettidia nigropunctatus*	0	1	0	0	1
*Culex quinquefasciatus*	128	96	11	7	242
*Culex tritaeniorhynchus*	21	15	4	4	44
*Culex vishnui*	64	82	73	80	299
*Culex gelidus*	0	2	0	1	3
*Culex fuscocephalus*	7	32	0	0	39
*Culex sitiens*	0	1	0	0	1
*Culex annulus*	3	7	1	2	13
*Mansonia uniformis*	66	101	28	22	217
*Mansonia dives*	90	164	37	32	323
*Anopheles nigerrimus*	14	20	31	34	99

**Table-3:** The relative abundance, species frequency, and dominance number of mosquitoes caught in Hulu Sungai Utara district with human landing catch method.

Mosquito species	Human landing collection		

	Relative abundance (%)	Species frequency	Dominance numbers
*Aedes aegypti*	0.3	0.5	0.2
*Aedes albopictus*	0.6	0.5	0.3
*Coquelettidia crassipes*	1.4	1.0	1.4
*Coquelettidia nigropunctatus*	0.1	0.3	0.0
*Culex quinquefasciatus*	18.5	1.0	18.5
*Culex tritaeniorhynchus*	3.4	1.0	3.4
*Culex vishnui*	22.8	1.0	22.8
*Culex gelidus*	0.2	0.5	0.1
*Culex fuscocephalus*	3.0	0.8	2.2
*Culex sitiens*	0.1	0.3	0.0
*Culex annulus*	1.0	1.0	1.0
*Mansonia uniformis*	16.6	1.0	16.6
*Mansonia dives*	24.6	1.0	24.6
*Anopheles nigerrimus*	7.6	1.0	7.5

### Dilation of mosquitoes

The captured mosquitoes were subjected to ovarian surgery to determine whether they had ever laid eggs (parous) or not (nulliparous). *M. uniformis* and *M. dives* were the most common mosquitoes found to be parous (Table-4).

**Table-4:** Number of dilation of mosquitoes caught in Hulu Sungai Utara district with human landing catch method.

Mosquito species	Pihaung village		Banjang village	
	
	Nulliparous	Parous	Nulliparous	Parous
*Aedes aegypti*	2	1	1	0
*Aedes albopictus*	5	3	0	0
*Coquelettidia crassipes*	10	7	0	0
*Coquelettidia nigropunctatus*	1	0	0	0
*Culex quinquefasciatus*	114	110	7	11
*Culex tritaeniorhynchus*	14	22	4	4
*Culex vishnui*	51	95	62	91
*Culex gelidus*	2	0	1	0
*Culex fuscocephalus*	17	22	0	0
*Culex sitiens*	0	1	0	0
*Culex annulus*	8	2	3	0
*Mansonia uniformis*	54	200	17	52
*Mansonia dives*	65	102	23	27
*Anopheles nigerrimus*	13	21	25	40
Total	356	586	143	248

### Density and periodicity of *M. dives* and *M. uniformis*

The man-hour density was high at 19.00-22.00 for *M. dives* and 18.00-20.00 for *M. uniformis*. Regarding their activities, the two mosquitoes sucked blood throughout the night, except at 24.00-01.00 for *M. uniformis* (Table-5).

**Table-5:** The man-hour density of *Mansonia dives* and *Mansonia uniformis*.

Mosquito collecting time	*Mansonia dives*		*Mansonia uniformis*	
	
	Indoors	Outhoors	Total	Indoors	Outdoors	Total
18.00-19.00	6.2	2.2	8.4	12.9	19.1	32.0
19.00-20.00	5.8	14.7	20.4	6.7	14.7	21.3
20.00-21.00	6.2	13.8	20.0	6.7	5.3	12.0
21.00-22.00	9.3	12.0	21.3	4.0	4.0	8.0
22.00-23.00	4.4	8.9	13.3	3.1	1.3	4.4
23.00-24.00	4.0	4.4	8.4	1.3	1.3	2.7
24.00-01.00	4.4	7.6	12.0	0.4	0.0	0.4
01.00-02.00	4.0	6.2	10.2	1.8	4.4	6.2
02.00-03.00	3.6	4.4	8.0	1.8	0.4	2.2
03.00-04.00	3.6	7.6	11.1	1.3	1.3	2.7
04.00-05.00	1.8	2.2	4.0	1.3	1.3	2.7
05.00-06.00	3.1	3.1	6.2	0.4	1.3	1.8

### Behaviors of *M. dives* and *M. uniformis*

**Figure-2:**
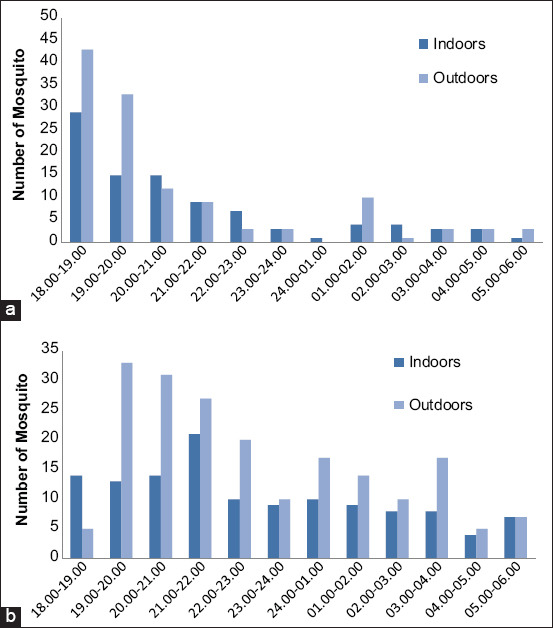
Periodicity of *Mansonia uniformis* (a) and *Mansonia dives* (b) from Hulu Sungai Utara District.

The blood-sucking activity of *M. uniformis* occurred at dusk, started to decline until 01:00, and then appeared again at 02.00 outside the house. On the other hand, *M. dives* was active at 19.00-21.00; its activity then decreased and appeared again at 01.00-04.00 (Figure-2).

### PCR analysis for the detection of microfilariae in mosquitoes

Table-6 shows several species of mosquitoes based on identification conducted at the capture location. A total of 802 mosquitoes were collected in HSU district, which were divided into 23 microtubes with different number of mosquitoes. The examined one species causing filariasis in HSU district. Based on PCR detection, six species were found in HSU district. A total of 311 *M. uniformis* were collected in 13 microtubes. One tube was positive for *W. bancrofti*. Therefore, the estimated infection and infectivity rates were each 0.3%.

**Table-6:** Mosquito species was found positive filaria DNA.

Mosquitos species	The number of mosquitoes test	The number of microtube test	The number of positive microtube	*Wuchereria bancrofti*	*Brugia* spp.
*Mansonia uniformis*	311	13	1	+	-
*Culex vishnui*	184	7	0	-	-
*Anopheles nigerrimus*	32	2	0	-	-
*Culex quinquefasciatus*	46	2	0	-	-
*Mansonia dives*	188	7	0	-	-
*Anopheles nigerrimus*	41	2	0	-	-

## Discussion

Based on the Shannon–Wiener diversity index, the diversity of mosquitoes in HSU district was found to be low (0.201-<1). The diversity of the genera *Culex* and *Mansonia* was more dominant than that of other genera. It has been reported that the presence of insects can be used as an indicator of ecosystem balance [16]. When the diversity of insects is high in an ecosystem, it indicates that the ecosystem environment is balanced or stable. Such high insect diversity will enable the normal running of the food web process, and vice versa, that is, low insect diversity in the ecosystem indicates an unbalanced and unstable ecosystem [17,18].

A total of 14 types of mosquitoes belonging to five genera were found in this study. All the 1311 mosquitoes captured in HSU district were identified and examined using PCR techniques. It was found that *M. uniformis* mosquito species contained the infective larvae of *W. bancrofti*, but several other species that were captured were also known as filariasis vectors, such as *C. quinquefasciatus* that can transmit urban-type *W. bancrofti* [19,20] and *M. dives* that can transmit *B. malayi* [21].

*M. uniformis* and *M. dives* mosquitoes are distributed throughout Kalimantan. *M. uniformis* has a blood-sucking behavior and is generally found outside the home, which is consistent with its exophagic and outdoor resting behavior in other areas of Kalimantan [22]. This behavior of *M. uniformis* indicates that human activities performed outside the home at night could potentially increase the risk of filariasis transmission. The lifespan of *M. uniformis* and *M. dives* mosquitoes in nature can reach up to 30 days under sufficient rainfall conditions [23]. This allows the occurrence of several gonotrophic cycles of *M. uniformis* and *M. dives*, and the filarial larvae take 8–12 days to become infective (L3) [24]. Mosquitoes can act as vectors of disease if they come into direct contact with the host which contain disease agents, the mosquito population is more dominant than other animals, and have longevity. [25].

Filariasis transmission is affected by several factors, including the presence of microfilaria-positive sufferers, the density of the infectious vector, the community behavior, and the ecological factors that affect vector density [26]. Regarding the characteristics of the research areas, they are wetlands in the form of swamps, with the livelihood of the population being dependent on agricultural land overgrown with rice plants. Rice itself is a potential habitat for *Mansonia* spp., which is a vector of filariasis in several areas in Kalimantan [22]. The larvae of *Mansonia* species live in permanent waters associated with aquatic plants such as rice, lotus, and kale that have roots that are used to attach the siphon to obtain oxygen from the air cells for respiration. The larvae have a sharp siphon to attach to plants [27]. In addition to filariasis-type *B. malayi, Mansonia* spp. is known to be a vector for Ross River virus, Murray Valley encephalitis virus, Kunjin virus, Edge Hill virus, and Rift Valley fever virus [28]. Several species of *Mansonia* have been reported as vectors in Kalimantan, namely, *M. bonneau* (Diptera: Culicidae) as the primary vector for filariasis caused by non-periodic *B. malayi* in the indigenous Dayak areas of East Kalimantan [29] and *M. uniformis* as the swamp-type filariasis vector in Batala District, South Kalimantan*. M. uniformis*, *M. dives*, and *M. bonneau* are the primary vectors of subperiodic *B. malayi* transmission [30] and are periodically nocturnal in some areas of South Thailand and Nakhon Si Thammarat, Narathiwat Surat Thani, Pattani, Phattalung, and Yala Province, and the secondary vectors are *M. dives*, *Mansonia annulata*, and *Mansonia annulifera* [31].

Kalimantan is known as a *B. malayi* endemic area, and it has been reported that *B. malayi* causes filariasis in 70% of the population in Indonesia [32]. It is also a parasite in primates, including humans and cats. Sheathed microfilariae generally occur nocturnally in the peripheral circulation, but there are also subperiodic strains. The nocturnal strains are host-specific, infecting humans exclusively, whereas the subperiodic strains infect not only humans but also cats, ape monkeys, and leaf monkeys [29]. The present study showed a different result of *W. bancrofti* positivity in *M. uniformis* in this district. A similar study conducted in Ghana also reported the presence of *W. bancrofti* in several *M. uniformis* and *M. africana* species [33]. That study also reported that an infective bite could transmit several parasites that could establish an active infection [33]. Almost all *W. bancrofti* parasites were found to be in the infected phase inside *Mansonia* spp. [34], which might explain the presence of infection in this province.

Preventive measures that can be implemented consist of education (counseling), vector identification (time and place of bite), vector control (changes in environmental construction), and treatments that can be provided on a mass or individual basis [35]. The biodiversity of mosquitoes is important to strengthen the current defective mosquito control approaches, and in-depth research on vector pathogen systems and vector biology could extend the development of enhanced vector harness control strategies. Application of these emerging concepts will ultimately offer a synergistic approach that will not only accelerate the elimination of lymphatic filariasis but also support its eradication in the future [36].

## Conclusion

This study has demonstrated the highest diversity of mosquitoes and that the transmission system could be more complex than expected in some areas in HSU district. *M. uniformis* species are apparently important vectors, although other species could also be other potential vectors of filariasis. Vector eradication is one component of elimination strategies in this subregion and can, therefore, have critical implications. It is essential to investigate the distribution, biology, and importance of vectors and also evaluate local vector control, if necessary, for this purpose.

## Authors’ Contributions

MRR and NR: Designed the study. MRR and BH: Conducted the field survey. MRR: Collected, dissected, and did identification of the mosquito samples. HK: Analysis of data. MRR, DP, HK, and BH: Drafted the manuscript. DP: PCR analysis. All authors revised, read, and approved the final manuscript.
